# Longitudinal assessment of renal function after lung transplantation for cystic fibrosis: transition from post-operative acute kidney injury to acute kidney disease and chronic kidney failure

**DOI:** 10.1007/s40620-022-01392-z

**Published:** 2022-07-15

**Authors:** Vittorio Scaravilli, Alessandra Merrino, Francesca Bichi, Fabiana Madotto, Letizia Corinna Morlacchi, Mario Nosotti, Alfredo Lissoni, Lorenzo Rosso, Francesco Blasi, Antonio Pesenti, Alberto Zanella, Giuseppe Castellano, Giacomo Grasselli

**Affiliations:** 1 Department of Anesthesia, Critical Care and Emergency, Fondazione IRCCS Ca’ Granda - Ospedale Maggiore Policlinico, Via F. Sforza 35, 20122 Milan, MI Italy; 2 Department of Biomedical, Surgical and Dental Sciences, University of Milan, Milan, MI Italy; 3 Department of Pathophysiology and Transplantation, University of Milan, Milan, MI Italy; 4 Department of Internal Medicine, Respiratory Unit and Cystic Fibrosis Center, Fondazione IRCCS Ca’ Granda - Ospedale Maggiore Policlinico, Milan, MI Italy; 5 Thoracic Surgery and Lung Transplant Unit, Fondazione IRCCS Ca’ Granda - Ospedale Maggiore Policlinico, Milan, MI Italy; 6 Dialysis and Renal Transplant Unit, Fondazione IRCCS Ca’ Granda - Ospedale Maggiore Policlinico, Milan, MI Italy; 7 Department of Clinical Sciences and Community Health, University of Milan, Milan, MI Italy

**Keywords:** Lung transplantation, Cystic fibrosis, Acute kidney injury, Acute kidney disease, Chronic kidney disease

## Abstract

**Introduction:**

The clinical trajectory of post-operative acute kidney injury (AKI) following lung transplantation for cystic fibrosis is unknown.

**Methods:**

Incidence and risk factors for post-operative AKI, acute kidney disease (AKD) and chronic kidney disease (CKD) were retrospectively analyzed in cystic fibrosis patients undergoing lung transplantation. Logistic regressions, Chi-square, Cuzick rank tests, and Cox-proportional hazard models were used.

**Results:**

Eighty-three patients were included. Creatinine peaked 3[2–4] days after transplantation, with 15(18%), 15(18%), and 20(24%) patients having post-operative AKI stages 1, 2, and 3, while 15(18%), 19(23%) and 10(12%) developed AKD stage 1, stage 2 and 3, respectively. Higher AKI stage was associated with worsening AKD (*p* = 0.009) and CKD (*p* = 0.015) stages. Of the 50 patients with AKI, 32(66%) transitioned to AKD stage > 0, and then 27 (56%) to CKD stage > 1. Female sex, extracorporeal membrane oxygenation support as a bridge to lung transplant and at the end of the surgery, the use of intraoperative blood components, and cold-ischemia time were associated with increased risk of post-operative AKI and AKD. Higher AKI stage prolonged invasive mechanical ventilation (*p* = 0.0001), ICU stay (*p* = 0.0001), and hospital stay (*p* = 0.0001), and increased the incidence of primary graft dysfunction (*p* = 0.035). Both AKI and AKD stages > 2 worsened long-term survival with risk ratios of 3.71 (1.34–10.2), *p* = 0.0131 and 2.65(1.02–6.87), *p* = 0.0443, respectively.

**Discussion:**

AKI is frequent in cystic fibrosis patients undergoing lung transplantation, it often evolves to AKD and to chronic kidney disease, thereby worsening short- and long-term outcomes.

**Graphical abstract:**

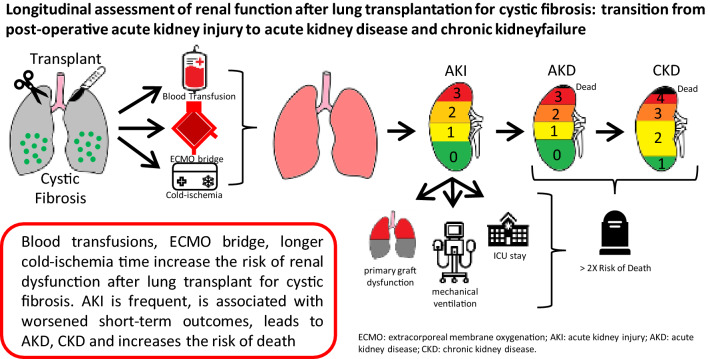

**Supplementary Information:**

The online version contains supplementary material available at 10.1007/s40620-022-01392-z.

## Introduction

Cystic fibrosis is an autosomal recessive inherited disease leading to progressive restrictive respiratory failure [1]. Bilateral lung transplantation provides a survival benefit in these patients [2]. Cystic fibrosis per se is not associated with impaired renal function [3]. Nevertheless, cystic fibrosis patients undergoing lung transplantation have a high risk of postoperative acute kidney injury (AKI) [4]. Indeed, in the immediate postoperative period, AKI occurs in up to two-thirds of transplanted patients, with 5–13% needing renal replacement therapy (RRT) and associated mortality ranging from 13 to 50%. In the long term, these patients frequently develop chronic kidney disease (CKD), with an overall incidence of 25% [4]. AKI and CKD are increasingly recognized as related entities, often representing a disease continuum [5]. In fact, the term acute kidney disease (AKD) has been introduced to describe the transition from AKI to CKD [6], representing a critical window where intervention and appropriate risk exposure management might mitigate this ominous evolution.

Few studies have evaluated the perioperative risk factors associated with AKI after lung transplantation [7–9] for cystic fibrosis. Moreover, to our knowledge, no study has described the transition from AKI to AKD and its final evolution in CKD in lung transplantation recipients, let alone in cystic fibrosis patients.

With this retrospective observational single-center cohort study, we performed a longitudinal assessment of the renal function of adult cystic fibrosis patients undergoing lung transplantation at an Italian tertiary referral center. We aimed at (1) describing the clinical trajectory of renal function, from post-operative AKI to AKD and CKD; (2) assessing the short- and long-term outcomes of AKI and AKD, and (3) investigating possible risk factors associated with post-operative AKI and AKD.

## Methods

### Ethical disclosures

The study was approved by the Institutional Ethics Committee and registered at clinicaltrials.gov with identifier NCT05001841. Informed consent was waived. The clinical and research activities reported are consistent with the Principles of the Declaration of Istanbul as per the “Declaration of Istanbul on Organ Trafficking and Transplant Tourism”.

### Study design

We performed a retrospective analysis of medical records of all consecutive cystic fibrosis patients who underwent lung transplantation at our Institution (Fondazione IRCCS Ca’ Granda—Ospedale Maggiore Policlinico, Milano, Italy) from January 2013 to December 2018. Exclusion criteria were: (1) single lung transplantation; (2) re-transplantation; (3) missing medical records. A detailed description of the management of lung transplantation at our Institution can be found in previous reports [10–12] and in the Online Supplement (Additional Methods).

### Measurements

Serum creatinine concentration (sCr) was recorded at the following time points: at enlistment; immediately before lung transplantation (i.e., 6 hours before surgery commencement); each day during the hospital stay until hospital discharge; at 90-day follow-up; at 1-year follow-up. Moreover, at the same time-points, we assessed the need for RRT. Thus, according to Kidney Disease Improving Global Outcomes (KDIGO) criteria for AKI [13], AKD [6], and CKD [13], patients were classified as having: (a) *pre-operative CKD*; (b) *post-operative AKI* (i.e., stage 1, stage 2, and stage 3 if increases of 1.5–1.9 times, 2–2.9 times, and > 3 times from the pre-operative creatinine were measured during hospital stay); (c) *post-operative AKD* stage 1, stage 2, and stage 3 if increases of 1.5–1.9 times, 2–2.9 times, and > 3 times sCr (or need for dialysis) from the pre-operative creatinine were measured at the 90-day follow-up visit; (d) *1-year follow-up CKD*. Notably, CKD stage 1, stage 2, stage 3, stage 4, and stage 5 was diagnosed if estimated glomerular filtration rate (eGFR), as calculated according to the 2021 CKD-EPI Creatinine formula [14], was higher than 90 mL/min/1.73 m^2^, between 90 and 60 mL/min/1.73 m^2^, 30-to-59 mL/min/1.73 m^2^, 30–15 mL/min/1.73 m^2^, and < 15 mL/min/1.73 m^2^ (or need for dialysis or kidney transplant), respectively (see Additional Methods for CKD staging).

The following data were collected at the time of enlistment for lung transplantation: age, gender, body mass index (BMI), co-morbidities, lung allocation score, need for extracorporeal membrane oxygenation (ECMO) bridge to lung transplantation; occurrence of pulmonary arterial hypertension and impaired right ventricular function. The following intra-operative data were collected: use of ECMO for lung transplantation and the need for ECMO at the end of surgery; the use of blood components and relative amount. The following donor data were collected: type of donor; OTO score [15] (see Online Supplement, Additional Methods for description); warm-ischemia times; cold-ischemia times; use of *ex-vivo* lung perfusion. The following outcomes were collected: length of invasive mechanical ventilation; primary graft dysfunction [16] grade 72 h after reperfusion; intensive care unit (ICU) length of stay (LOS); hospital LOS; survival at 31st May, 2021.

### Statistics

Data were reported as the median [first-third quartile] and number of events (percentage of the subgroup) for continuous and categorical variables, respectively. Patients with pre-operative CKD stage > 3 at enlistment were excluded from the analysis of the possible risk factors for perioperative AKI. Variations of sCr over time were assessed using a generalized linear model, with subjects as random effects, time-points as covariates and patient cohorts (i.e., CKD stage > 2 vs. CKD ≤ 2) as fixed effect. Comparison between patient cohorts (e.g., AKI stage ≥ 1 vs. AKI stage = 0) was performed with logistic regression or the Chi-squared test, as appropriate. For binary outcome measures, odds ratios (ORs) and associated 95% likelihood ratio-based confidence intervals were calculated. Stepwise logistic regression analysis with forward selection was applied in order to identify predictors for AKI. A critical level of 5 in the variance inflation factor (VIF) was used to identify independent variables affected by multicollinearity in order to exclude them from the selection procedure (see Online Supplement for further details). The Cuzick rank test was utilized to trend in short term outcomes across AKI stages. Kaplan–Meier survival curve analysis was used with the log-rank test for comparison of patient survival. Cox-proportional hazard models were utilized to evaluate the effects of perioperative AKI and post-operative AKD upon survival. Clinically meaningful variables were included in the model as covariates, based on available literature (i.e., age [17], BMI [18], diabetes [19], gender [20], pulmonary hypertension [21], use of intraoperative ECMO [12], lung allocation score [22], and primary graft dysfunction [23]). Observations were right-censored. All statistical tests were 2-tailed, and statistical significance was accepted at P < 0.05. The JMP® pro 14.0 (SAS, Cary, NC) was utilized.

## Results

### Patients’ characteristics

From January 2013 to December 2018, 85 consecutive cystic fibrosis patients underwent double lung transplantation at our Institution. Two of these patients were re-transplants, and hence, 83 cystic fibrosis patients were included in the study.

Two (2·4%) patients had CKD stage > 3 at enlistment, and they were thus excluded from the analyses. The clinical course of these two patients is described in-depth in the Online Supplement (Additional Results). Notably, both patients suffered AKI, AKD, and CKD stage 5, and ultimately died prior to the 1-year follow-up period.

The patients' clinical characteristics at enrollment are shown in Table S1 (see Online Supplement, Additional Results). At enlistment the patients showed normal sCr (i.e., 0·6 [0·47–0·76]) mg/dL with eGFR (i.e., 127 [117–134]) mL/min/1·73 m^2^). sCr peaked at a median of 3 [2–4] days after lung transplantation (see Online Supplement, Additional Results, Figure S1).

### Clinical trajectory of renal function

**Fig. 1:**
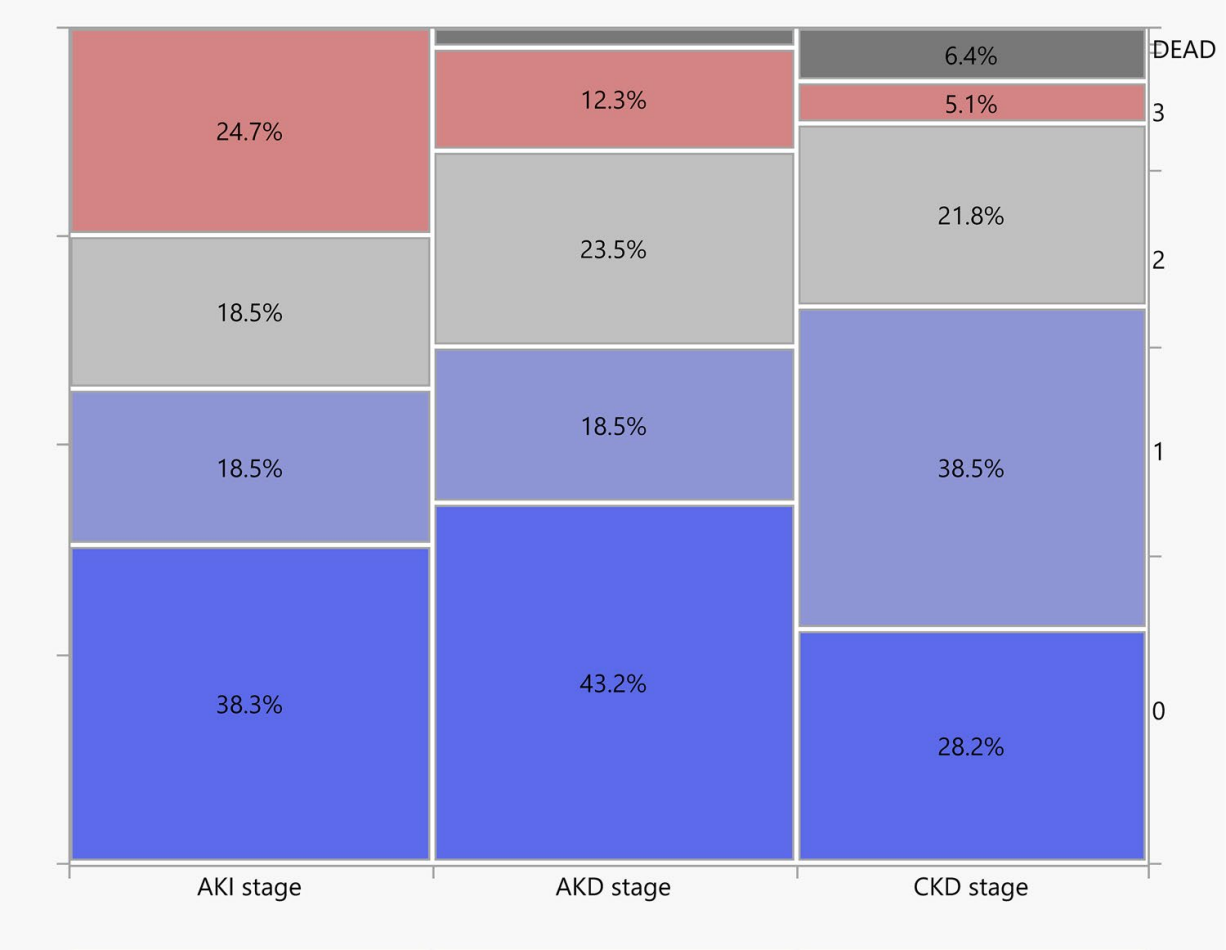
Overall incidence of renal dysfunction. *AKI* acute kidney injury, *AKD* acute kidney disease, *CKD* chronic kidney disease

Figure 1 shows the overall incidence of renal dysfunction. Fifteen (18%), 15 (18%), and 20 (24·7%) patients developed post-operative AKI stage 1, AKI stage 2, and AKI stage 3, respectively, with 7 (8·6%) needing renal replacement therapy during the ICU stay. At the 90-day follow-up visit, 2 patients had died, while 15 (18%), 19 (23%) and 10 (12%) developed AKD stage 1, stage 2 and 3, respectively. At 1-year follow-up, among 76 survivors, only 22 (28.2%) of the patients showed no renal injury (CKD stage 1), with up to 27% of the patients having CKD stage > 2.

**Fig. 2:**
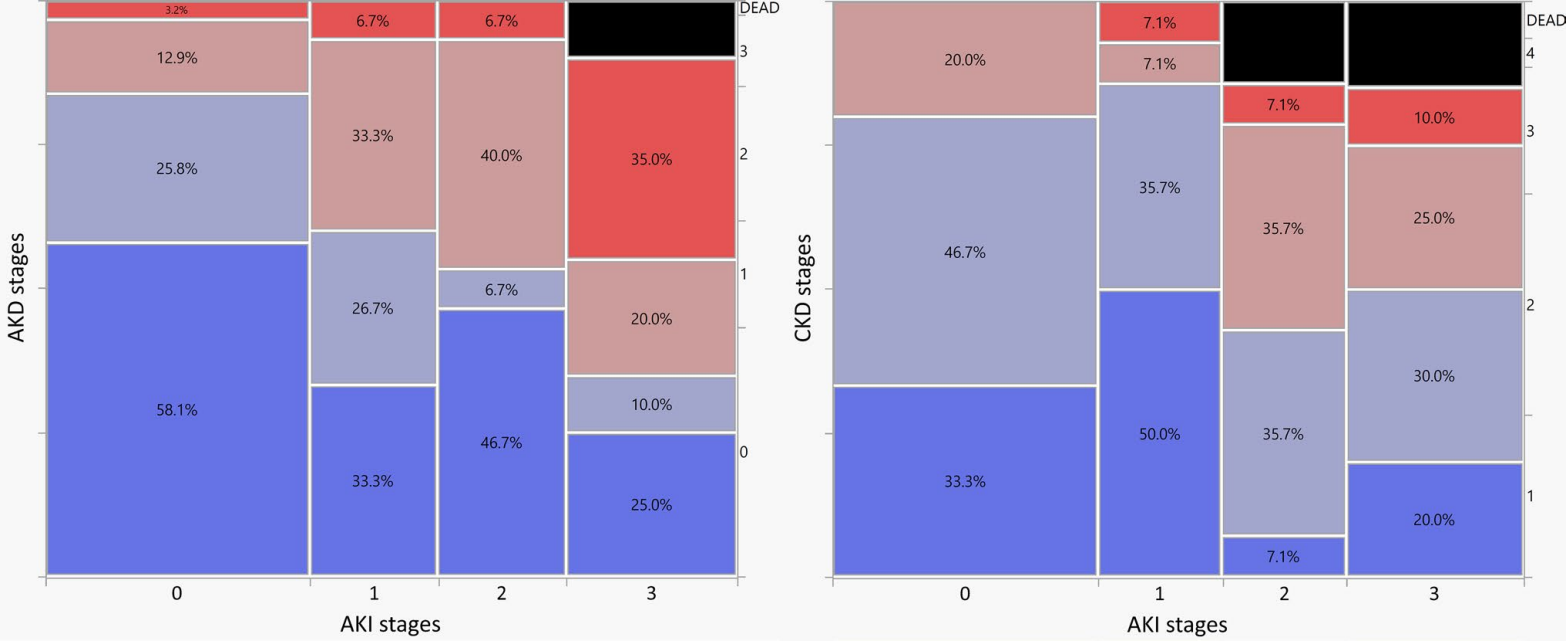
Incidence of acute kidney injury and impact on long-term chronic kidney disease. **A** Mosaic plot of the incidence of acute kidney disease vs. acute kidney injury during ICU stay. *AKI* acute kidney injury, *ICU* intensive care unit. The width, height, and area of the rectangles are proportional to the number of patients per cohort, the frequency of acute kidney injury stage, and the cell frequencies of the contingency table. *AKI* acute kidney injury, *AKD* acute kidney disease. **B** Mosaic plot of the incidence of 1-year follow-up chronic kidney disease vs. acute kidney injury during ICU stay. *AKI* acute kidney injury, *ICU* intensive care unit. The width, height, and area of the rectangles are proportional to the number of patients per cohort, the frequency of acute kidney injury stage, and the cell frequencies of the contingency table. *AKI* acute kidney injury, *CKD* chronic kidney disease

Figure 2 shows the association between AKI, AKD, and CKD. Higher AKI stage severity was associated with worsening AKD stage (*p* = 0·009) and with 1-year follow-up CKD stage (*p* = 0·035) severity. Of the 50 patients with AKI during ICU stay, 32 (66%) transitioned to AKD stage > 0, and then 24 (48%) to CKD stage > 1, with only 12 (24%) returning to normal renal function at 1-year follow-up.

**Fig. 3:**
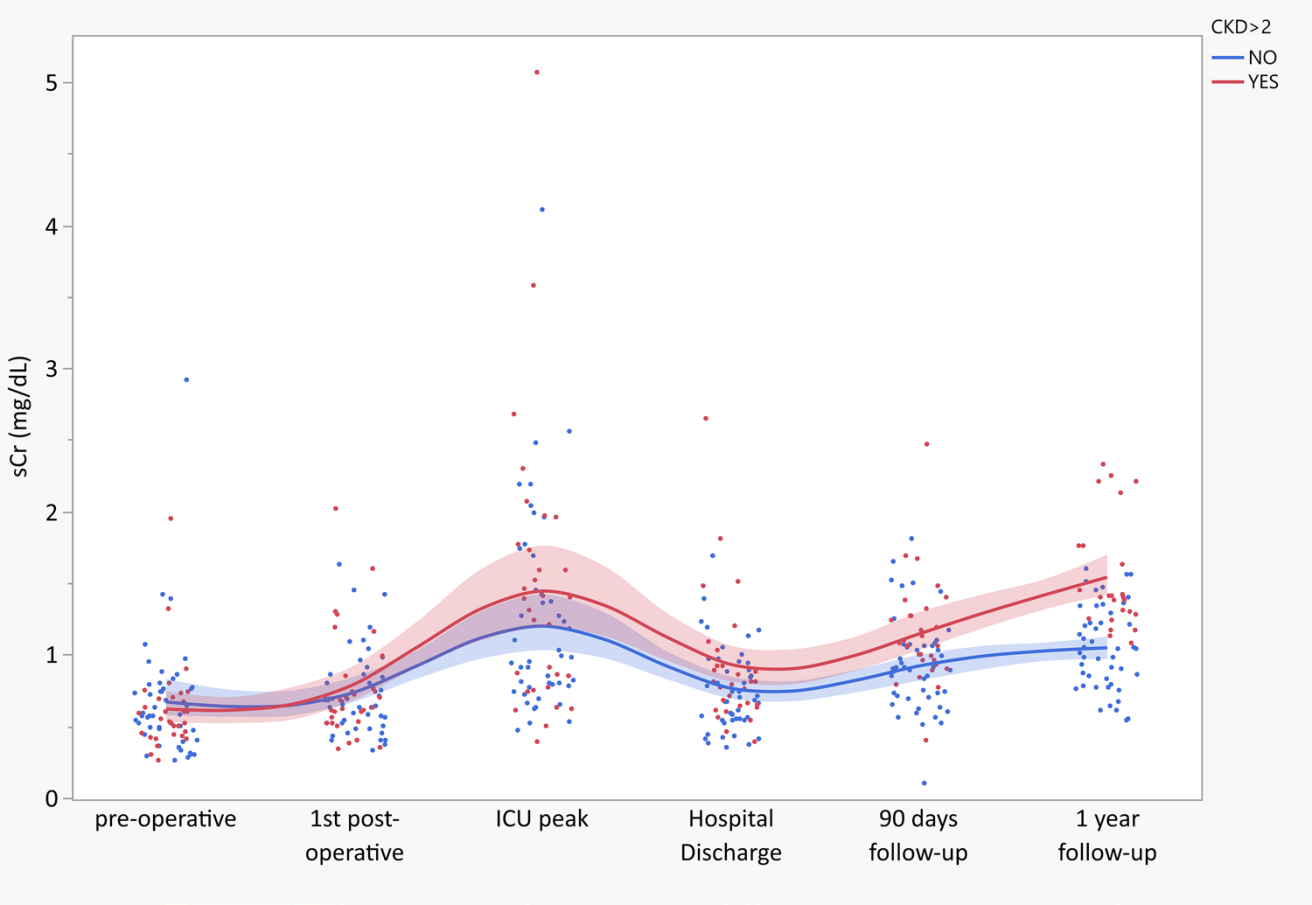
Serum Creatinine trajectory over time. *sCR* serum creatinine, *ICU* intensive care unit, *CKD* chronic kidney disease. Blue and red dots represent sCr concentration of patients with CKD stage ≤ 2 and CKD stage > 2, respectively. Blue and red lines represent cubic spline interpolation (*λ* = 0.05) of sCr of patients with CKD stage ≤ 2 and CKD stage > 2, respectively, with associated 95% confidence intervals as the colored areas

Figure 3 describes the transition from AKI to AKD and CKD. Patients with CKD stage > 2 (*n* = 31, 39·5%) showed sCr statistically significantly higher at the 90-day follow-up visit (*p* = 0·007), with similar sCr at baseline, during the ICU stay, and at hospital discharge.

### Outcomes of renal dysfunction

Short-term outcomes are shown in Figure S2. The Cuzick test showed a statistically significant trend between higher AKI stage and longer duration of invasive mechanical ventilation (*p* = 0·0001), ICU length of stay (*p* = 0·0001), and hospital length of stay (*p* = 0·0001). Moreover, we observed a strict association between the primary graft dysfunction stage and AKI stage during the ICU stay (*p* = 0·035), see Figure S3.

**Fig. 4:**
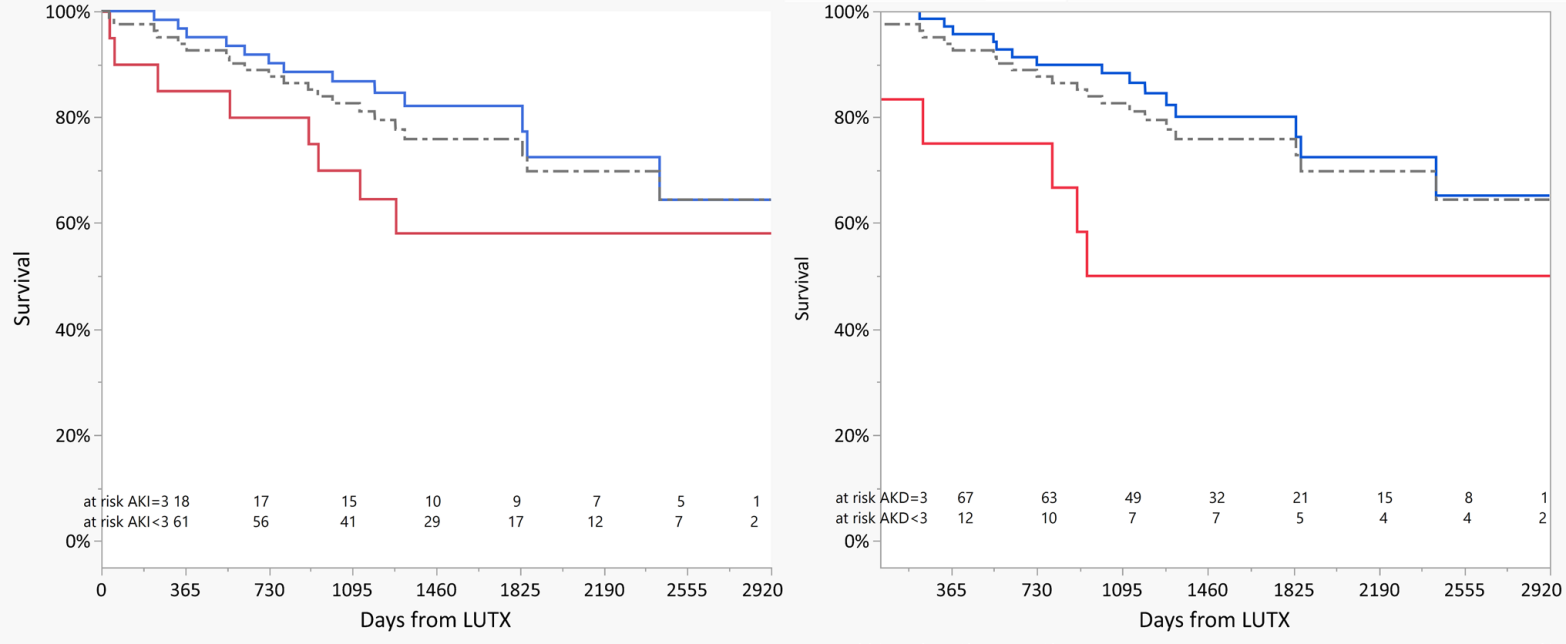
Probability of survival. Left panel: Kaplan − Meier estimates of the unadjusted cumulative probability of survival following acute kidney injury (AKI). Red and blue lines represent patients with AKI stage = 3 and AKI stage < 3, respectively. Right panel: Kaplan − Meier estimates of the unadjusted cumulative probability of survival following acute kidney disease (AKD). Red and blue lines represent patients with AKD stage = 3 and AKD stage < 3, respectively. Grey dotted lines represent the overall population

Both post-operative AKI and AKD worsened long-term survival (see Fig. 4), with increased risk ratios for death of 3.71 (1.34–10.2), p = 0.0131 and 2.65 (1.02–6.87), *p* = 0.0443 for AKI stage 3 and AKD stage 3, respectively. Moreover, the multivariable Cox-proportional hazard model showed that AKI stage ≥ 1 was an independent risk factor for long-term survival (*p* = 0.022, HR 3.46 (1.08–11.1)) (see Table S2, Online Supplement, Additional Results). Such associations were not observed for any AKD stage.

### Risk factors for renal dysfunction

The pre-operative, intra-operative recipient, and donor characteristics associated with increased risk of post-operative AKI stage ≥ 1 and AKD stage > 1 are shown in Tables S3 and S4. The need for prolonged ECMO support at the end of the surgical procedure, the need for ECMO bridge to lung transplant, the use of intraoperative blood components and red blood cell concentrates, and cold-ischemia time were associated with increased risk of both post-operative AKI and 90-day AKD. Stepwise logistic regression analysis identified male sex (*p* = 0·013, OR 0·28 (1·10–0·76) and longer cold-ischemia time (*p* = 0·010, OR 1·15 (1·03–1·28)) as independently reducing and increasing the risk of AKI, respectively.

## Discussion

This retrospective cohort study describes the renal function trajectory after double lung transplantation for cystic fibrosis. We observed that AKI frequently occurs (i.e., > 60%), worsens short-term outcomes, increases the risk of primary graft dysfunction, often evolves to AKD and then to chronic kidney disease, leading to an overall shortened survival.

Previous literature has described the incidence of AKI after lung transplantation [4, 7–9], showing an incidence of post-operative AKI of around 50%, with an associated almost three-fold risk of hospital death in large cohorts of adult patients undergoing lung transplantation for mixed indications. However, none of those cohort studies focused on cystic fibrosis patients. We focused our analysis on adult patients with cystic fibrosis for two reasons. First, our center is a tertiary referral center for cystic fibrosis. Second, compared to the general population of patients undergoing lung transplantation, cystic fibrosis patients are younger and have few non-cystic fibrosis-related comorbidities. Thus, targeting this homogeneous population may unbias the results from the multiple comorbidities that are common in other patient cohorts. Our patients were young, underweight, with almost no comorbidities, and were affected by life-limiting lung disease. Notably, enlistment and pre-operative renal function were almost normal. An obvious selection bias is present, given that patients with renal failure are rarely enlisted for lung transplantation.

In our population of adult cystic fibrosis patients undergoing lung transplantation, AKI occurred very often: only 37.3% showed no sign of renal damage, and 9% needed renal replacement therapy during their ICU stay. Furthermore, renal dysfunction was virtually absent on the first post-operative day and occurred at a median of 3 days after lung transplantation, as previously shown [7–9]. Then, in most patients, sCr returned to normal at hospital discharge, only to increase again at the 90-day follow up visit and even more so 1 year after lung transplantation. AKD occurred in a significant proportion (almost 60%) of patients after lung transplantation. Our analyses showed that greater severity of post-operative AKI was associated with subsequent worsening severity of AKD and finally CKD. Lastly, in the long term, both AKI and AKD were associated with worsened survival.

To the best of our knowledge, no previous study has assessed the incidence of AKD after lung transplantation.

Interestingly, the renal function trajectory of patients with long-term impaired renal function (i.e., CKD > 2) diverged from the rest of the cohort early on in the postoperative course and was clearly dichotomized at the 90-day follow-up visit, thus offering a fascinating window for possible early diagnosis and intervention.

The early occurrence of AKI, its persistence, and continuation into AKD suggest that intraoperative events may play an essential causative role in impairing renal function. We observed that variables associated with a more complicated and invasive surgical procedure (i.e., need for ECMO at the end of surgery, the use of intraoperative blood components, cold-ischemia time) increased AKI risk, consistently with previous works [8]. Even more, the same risk factors were valid for AKD, further underlining how AKI and AKD represent a clinical continuum. The finding that female patients are at higher risk of AKI after lung transplant is new, in contrast with previous literature. Further studies are necessary to assess whether this association is simply a consequence of unmeasured confounders or a causal association worth further and specific management. On the other hand, the association between cold-ischemia time and AKI is in line with previous evidence [24], and is reasonably associated with the more severe ischemia–reperfusion renal injury following the implant of lungs suffering longer ischemic times.

As per the association between ECMO bridge to lung transplantation with postoperative renal failure, it is reasonable to assume that ECMO bridge does not increase the risk of AKI per se, but rather that ECMO bridge represents a proxy for clinical compromise. The association between higher red blood cell transfusions and AKI (and AKD) risk following lung transplantation warrants further consideration. Indeed, red blood cell transfusions have proven to be an independent risk factor for renal failure in patients undergoing cardiac surgery by cardiopulmonary bypass [25, 26]. We do not have knowledge of prior studies showing this association in patients undergoing lung transplantation. Possibly, the pathophysiological pathways leading to AKI during cardiac surgery [27] (i.e., circulating free-iron mediated nephrotoxicity) may also play a role during lung transplantation, a surgical procedure that is similarly characterized by the frequent use of ECMO, the occurrence of hypotension, and bleeding. Nevertheless, the study design and cohort size allowed us to carry out a limited and exploratory multivariable inferential analysis. Thus, we cannot disentangle the impact of transfusions on renal function from possible other covariates (e.g., hypoxia, hypoperfusion, ECMO need). Therefore, further studies are necessary to confirm such association and to evaluate the role of blood-loss-minimizing techniques to control this modifiable risk factor for AKI and AKD.

In line with previous findings, we observed that AKI severely impacts clinically meaningful short-term outcomes. Indeed, higher AKI stages were associated with increased duration of invasive mechanical ventilation, ICU- and hospital length of stay. We confirm the impressive association of AKI with primary graft dysfunction. Such association was recently documented in a mixed-indication cohort of lung transplantation patients [27], including only 26 cystic fibrosis patients. Given the concurrence of AKI and primary graft dysfunction, we cannot infer any causality between the two, but several pathophysiological mechanisms may link them. On the one hand, treatment of AKI by liberal volume infusion may impair graft function. On the other hand, the treatment of primary graft dysfunction by prolonged mechanical ventilation, volume restriction, and enforced calcineurin immunosuppression may impair kidney function. A more holistic—and elegant—explanation may be that AKI and primary graft dysfunction share the same pathophysiology, being similarly triggered by ischemia–reperfusion following transplant. Such a hypothesis deserves further evaluation.

Finally, we observed that AKI affected mortality independently of several other essential covariates (i.e., age, gender, BMI, diabetes, lung allocation score, pulmonary hypertension, intraoperative ECMO, and primary graft dysfunction) and that the risk ratio for death at follow-up was more than three-fold for any AKI stage. These results align with the previous literature [28, 29].

Our study has several limitations. First, to assess the incidence of CKD we utilized the most recent iteration of the CKD-EPI calculation of GFR based on sCr [14], which in a population of patients suffering from depleted muscle mass might be particularly biased. In this cohort of patients, cystatin-C clearance-based calculation of GFR has proven more accurate [30], and thus future studies should base their analysis on equations obtained using cystatin-C clearance. Second, this is a study from a highly specialized unit including only patients with cystic fibrosis, thus limiting the generalizability of our results to the broader patient population. Third, despite being the largest study focusing on AKI following lung transplantation in cystic fibrosis patients, the number of patients was relatively small. Thus, the results of the multivariable inferential analysis of the risk factors for AKI must be considered as exploratory, and further more-populated studies are necessary to confirm these analyses. Finally, due to the study's retrospective design, we could not record several other important intraoperative factors that may have impacted post-operative renal function (e.g., hypotensive episodes, use of vasoactive agents). Thus, such variables should be assessed through a prospective observational study.

AKI is frequent in cystic fibrosis patients undergoing lung transplantation, it worsens short-term outcomes, increases the risk of primary graft dysfunction, often evolves to AKD and to chronic kidney disease, leading to overall shorter survival. Future studies are necessary to assess the pathophysiological pathways leading to AKI and AKD after lung transplantation and to test possible risk-mitigating clinical approaches.

## Supplementary Information

Below is the link to the electronic supplementary material.

Additional supporting information may be found online in the Supporting Information section (DOCX 668 kb)

## Data Availability

The complete anonymized dataset will be available upon request to the corresponding author.
