# Supplementary material for: Longitudinal assessment of renal function after lung transplantation for cystic fibrosis: transition from post-operative acute kidney injury to acute kidney disease and chronic kidney failure
Source: J Nephrol. 2022 Jul 15;35(7):1885–93. doi: 10.1007/s40620-022-01392-z (PMC9458565; doi:10.1007/s40620-022-01392-z)
Supplement: Supplementary file 1 — Additional supporting information may be found online in the Supporting Information section (DOCX 668 kb) [file 40620_2022_1392_MOESM1_ESM.docx]

**Online Supplement**

**Table of contents**

1. **Additional Methods**
2. Oto Score calculation
3. Primary graft dysfunction grade
4. Surgical Procedure
5. eGFR calculation and CKD staging
6. Stepwise logistic regression analysis
7. **Additional Results**
8. Table S1. Overall cohort patients' clinical characteristics
9. Clinical Course of the two patients with pre-operative CKD
10. Figure S1. Changes in serum creatinine concentration at the different time points.
11. Figure S2. Impact of acute kidney injury stage during ICU stay upon short-term outcomes.
12. Figure S3. Figure 4. Mosaic plot of the incidence of 72-hour primary graft dysfunction vs. acute kidney injury during ICU stay.
13. Table S2. Survival analysis.
14. Table S3. Risk factors for acute kidney injury.
15. Table S4. Risk factors for acute kidney disease.
16. **Additional Methods**
17. *Oto Score calculation*

A donor lung score was calculated as previously shown by Oto et al. (see Oto, Annals of Thoracic Surgery, 2007). For each donor, a score was obtained following the sum of each domain. For donors after cardiac death (n=4), the Oto score cannot be calculated.

| *Category* | *Stratification* | *Score* |
| --- | --- | --- |
| Age (years) | < 45 | 0 |
|  | 45-54 | 1 |
|  | 55-59 | 2 |
|  | ≥ 60 | 3 |
| Smoking history  (pack-years) | < 20 | 0 |
|  | 20-39 | 1 |
|  | 40-59 | 2 |
|  | ≥ 60 | 3 |
| Chest X-ray | Clear | 0 |
|  | Minor | 1 |
|  | Opacity ≤ 1 lobe | 2 |
|  | Opacity > 1 lobe | 3 |
| Secretions | None | 0 |
|  | Minor | 1 |
|  | Moderate | 2 |
|  | Major | 3 |
| PaO_2_/FIO_2_ | > 450 | 0 |
|  | 351-450 | 2 |
|  | 301-350 | 4 |
|  | ≤ 300 | 6 |

1. *Primary Graft Dysfunction Grading*

Primary graft dysfunction was evaluated following the latest ISHLT consensus (see Snell, Journal of Heart and Lung Transplant, 2017) 72 hours after reperfusion, and graded as follows:

| *Grade* | *Radiographic Sign* | *PaO_2_/FIO_2_* |
| --- | --- | --- |
| 0 | No | > 300 |
| 1 | Diffuse pulmonary edema | > 300 |
| 2 | Diffuse pulmonary edema | 200 to 300 |
| 3 | Diffuse pulmonary edema | < 200 |

1. *Surgical procedure*

The Lung transplantation (LUTX) team at Fondazione IRCCS Ca’ Granda - Ospedale Maggiore Policlinico is composed of two thoracic surgeons, one thoracic surgery fellow, a cardiac surgeon, two certified anesthesiologists, one thoracic anesthesia fellow, and one anesthesia fellow, a perfusionist, an operating-room nurse and one surgical-nurse.

After routine monitoring as per ASA guidelines, induction of general anesthesia with fentanyl (1-2 mcg/kg), midazolam (1-2 mg) and propofol (1-3 mg/kg), and muscle paralysis with rocuronium (0.6 mg/kg) is achieved. Patients are intubated with a large size single-lumen tube and undergo aggressive bronchoscopic toilet. Then, the single-lumen tube is substituted, and lung isolation is achieved with an appropriately sized left-sided double-lumen endotracheal tube under bronchoscopic guidance. General balanced anesthesia is maintained with fentanyl (1-2 mcg/kg/hr) and sevoflurane (end-tidal concentration 0.5%-1.0%), and muscle paralysis with sequential rocuronium boluses (0.15 mg/kg). Patients are monitored with invasive right radial artery cannulation, central venous catheterization of the right internal jugular vein, and oximetric pulmonary artery catheterization capable of cardiac output monitoring by means of thermodilution technique. Trans-esophageal echocardiography is implemented depending on the anesthesiologist’s preferences. A large bore (i.e., 7 Fr) catheter is introduced in the right antecubital vein and connected to a custom-made rapid infusion system capable of infusing up to 500 mL/min. Throughout the procedure, patients are mechanically ventilated in volume control mode, with FiO_2_ to maintain SpO_2_> 90%, and minute ventilation to limit hypercapnia and acidosis.

Antibiotic prophylaxis is provided following the indication of infectious disease specialists and pre-operative airway cultures. Immunosuppression comprises methylprednisolone 1000 mg and tacrolimus or basiliximab as per the patient’s renal function.

Patients with CF are treated with sequential bilateral LUTX, using bilateral anterolateral thoracotomy with a transverse sternotomy or two anterior thoracotomies, based on the anatomical characteristics of the patients. After lysis of pleural and mediastinal adhesions, the vascular structures and bronchi are isolated. Then the less perfused lung (as per pulmonary perfusion scintigraphy) is disconnected from the mechanical ventilation and allowed to deflate. The pulmonary artery is cross-clamped, and the first cross-clamping 10-minute test is performed: the surgical procedure is halted, hemodynamics are strictly monitored,and blood gas analyses are obtained every 2 minutes.

The veins, artery, and bronchus are sequentially resected. The graft is positioned in the thoracic cavity, and bronchial, arterial and venous anastomoses are created. The lung is thoroughly de-aired before vascular unclamping to avoid systemic air emboli; the first lung graft is slowly reperfused and connected to another mechanical ventilator. The graft is initially ventilated in pressure control mode with FiO_2_ 21%, PEEP of 10 cmH_2_O, RR of 4 bpm and plateau pressure of 25 cmH_2_O. A recruitment maneuver is applied to obtain complete lung inflation. Progressively, ventilation and oxygenation of the graft are increased to allow contralateral lung separation from ventilation. Particular attention is paid in limiting 1) FiO_2_ (i.e., < 50%); 2) driving pressures (i.e., < 15 cmH2O) and 3) de-recruitment (i.e., PEEP > 10 cmH_2_O) of the implanted graft. Then, contralateral native lung ventilation is interrupted, and the second pulmonary artery cross-clamping test is performed. Pneumonectomy of the second native lung and implantation of the second graft follows the procedure above. Finally, hemostasis is achieved, bilateral pleural drainage positioned, the thorax closed and the patient is transferred to the intensive care unit for follow-up.

The pulmonary artery cross-clamping test is performed to simulate the hemodynamic conditions occurring during pneumonectomy in a controlled -and reversible- fashion, allowing both the anesthesiologist to optimize hemodynamics and ventilation and, if necessary, the cardiac surgeon to implement central veno-arterial ECMO in a semi-elective condition. During the first pulmonary artery cross-clamping test (while the native lung is ventilated and perfused), in case of 1) pulmonary hypertension (i.e., systolic pulmonary artery pressure > 80 mmHg, 2) increase in PAPs > 50 mmHg associated with systemic hypotension (i.e., systolic arterial pressure < 60 mmHg) resistant to inotropic support; 3) major cardiac arrhythmias; 4) hypoxia (i.e., PaO_2_< 60 mmHg, despite increasing FiO_2_ up to 100% and optimizing PEEP); 5) respiratory acidosis (i.e., pH < 7.25 despite increasing minute ventilation), the test is interrupted and ECMO is implemented.

During the second pulmonary-artery cross-clamping (while the graft is ventilated and perfused), our policy is more protective towards hyperoxia and ventilator-induced lung injury. Thus, during the second test FiO_2_ is not increased > 50% and driving pressure is not increased > 15 cmH_2_O. If one of the mentioned conditions occurs, ECMO is implemented.

Throughout the entire surgical procedure, intractable hypoxemia, acidosis, and hemodynamic failure may occur at any given moment, but 1) single lung ventilation, 2) cross-clamping tests and 3) reperfusion of the grafts are the most critical time points. During the procure, in case of 1) pulmonary hypertension (i.e., systolic pulmonary artery pressure > 80 mmHg, 2) increase in PAPs > 50 mmHg associated with systemic hypotension (i.e., systolic arterial pressure < 60 mmHg) resistant to inotropic support; 3) major cardiac arrhythmias; 4) hypoxia (i.e., PaO_2_< 60 mmHg, despite increasing FiO_2_ up to 100% to the native lung or 50% to the grafts and optimizing PEEP); 5) respiratory acidosis (i.e., pH < 7.25 despite increasing minute ventilation, while guaranteed driving pressure to the grafts < 15 cmH_2_O), the procedure is briefly halted, and ECMO is implemented.

Our approach to intraoperative extracorporeal life support consists of central veno-arterial ECMO. After providing unfractionated heparin (i.e., 5000 UI) and possible further boluses to achieve an aPTT>40 seconds, the ascending aorta and right atrium are cannulated. Blood is drained via a centrifugal pump directly to a polypropylene membrane lung where blood is oxygenated, decarboxylated, warmed and then directed to the central venous circulation. Initially, blood flow is set to achieve around 50% of the patient’s cardiac output, and gas flow is set to maintain normocapnia and fraction of oxygen in the sweep gas flow to maintain SpO_2_> 95%. The extracorporeal circuit setting is dynamically modified during the procedure on the basis of the different surgical and anesthetic requirements, and mean arterial pressure is maintained > 60 mmHg by increases in extracorporeal blood flow, but complete blood drainage and emptying of the heart is avoided, and the opening of the aortic valve is always guaranteed.

At the end of the surgical procedure, prior to chest closure, patients undergoing VA-ECMO undergo a progressive de-escalation of extracorporeal support, consisting of: 1) reduction of blood flows down to 1 L/min; 2) reduction of extracorporeal FiO2 down to 21%. Hemodynamics are monitored and blood gas analyses are collected serially to verify that the patient does not experience: 1) hemodynamic failure/right ventricular failure; 2) hypoxemia; 3) acidosis and hypercapnia and/or need to ventilate with tidal volume > 6 mL/kg or driving pressure > 14 cmH2O. If hemodynamic failure is observed, central VA-ECMO is converted to peripheral VA support (i.e., femoral cannulations). In case hypoxemia and/or hypercapnia is observed, VV-ECMO (i.e., femoral-femoral cannulation) is instituted and prolonged in the post-operatory period.

No predefined standard management of blood components is applied, but patient-tailored transfusion management is carried out following blood gas analyses and point of care (POC) PT/aPTT tests, as well as thromboelastography, as per national guidelines (<http://www.siaarti.it/standardclinici/SIAARTI%20standard%20PBM.pdf>).

1. *eGFR calculation*

The estimated Glomerular Filtration Rate was calculated according to the 2021 CKD-EPI Creatinine formula (as per N Engl J Med. 2021 Nov 4;385(19):1737-1749).

Following the eGFR calculation, CKD stages were assigned to each patient, as per the following formula.
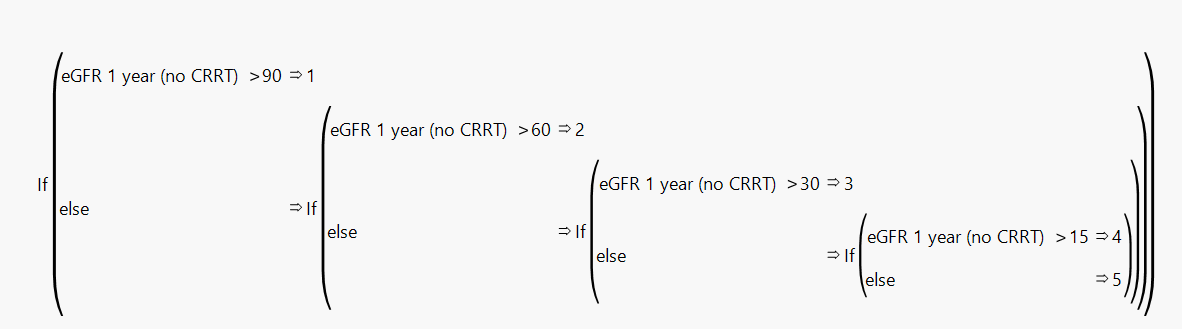
Moreover, patients in need of renal replacement therapy or enrolled for kidney transplant at follow-up were staged as CKD stage 5.

1. *Stepwise logistic regression analysis*

A stepwise logistic regression analysis was performed to evaluate possible independent risk factors for AKI. The analysis included patient enrollment and perioperative and donor clinical characteristics studied in the univariate logistic analysis. First, a missing data analysis was carried out as follows.

|  | **Missing data (%)** |
| --- | --- |
| **Enrollment** |  |
| Sex | 0 (0.00) |
| Age | 0 (0.00) |
| BMI | 0 (0.00) |
| Diabetes mellitus | 0 (0.00) |
| PAH | 2 (2.47) |
| FEdx | 8 (9.88) |
| eGFR | 0 (0.00) |
| Bridge to LUTX | 0 (0.00) |
| LAS | 0 (0.00) |
| **Perioperative** |  |
| Intraoperative ECMO | 0 (0.00) |
| Postoperative ECMO | 0 (0.00) |
| Blood components | 0 (0.00) |
| Red Blood Cells | 0 (0.00) |
| **Donor** |  |
| DBD donor | 0 (0.00) |
| Oto SCORE | 4 (4.94) |
| Total warm-ischemia time | 0 (0.00) |
| Total cold-ischemia time | 0 (0.00) |
| EVLP (%) | 0 (0.00) |
|  |  |
| **N without missing data** | **69 (85.19)** |

Only FEdx (i.e., the right ventricular ejection fraction) had > 5% missing data, and was thus removed from the analysis.

Then, the correlation between the covariates was assessed, as follows.


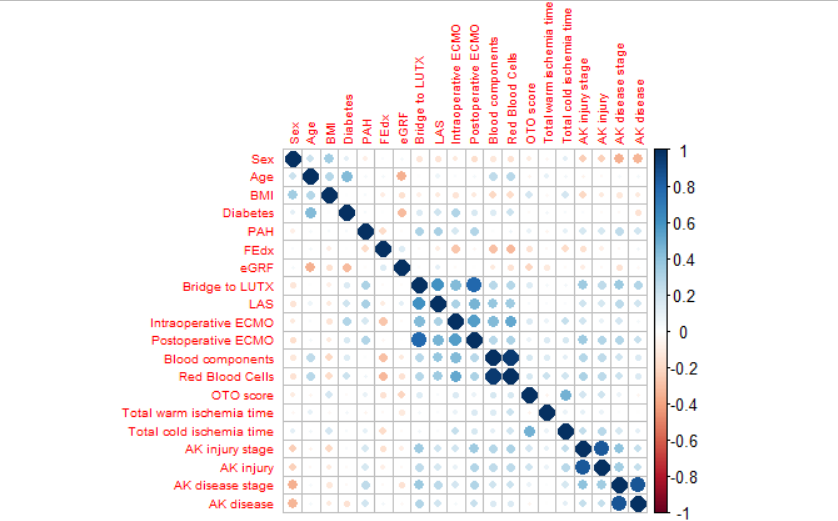
The correlation matrix showed that several covariates had elevated VIF (variation inflation factor), and thus were correlated or collinear. As an example, as expected, the total amount of blood components and the number of red blood cell concentrates had VIF >5. Similarly, a high LAS was correlated with the need for ECMO as a bridge to lung transplant, since the most severe patients have high LAS and are more likely to require ECMO support prior to lung transplantation. Moreover, all the patients bridged to transplant with ECMO continued ECMO support during the transplant, and in many cases needed ECMO at the end of surgery.

1. **Additional Results**
2. **Table S1.** Overall* cohort patients' clinical characteristics (n=81)

|  | **Clinical Characteristic** |  |
| --- | --- | --- |
| Enlistment | Age (years) | 30 [23 - 37] |
|  | Sex (male) | 40 (49.4%) |
|  | BMI (kg/m2) | 19.5 [18.3 - 21.5] |
|  | Diabetes (%) | 45 (55.6%) |
|  | PAH (%) | 17 (21.5%) |
|  | FEdx < 40% (%) | 14 (18.9%) |
|  | eGFR (mL/min/1.73 m^2^) | 132.4 [98.9 - 155.4] |
|  | Waiting List (days) | 145 [58 - 328] |
|  | Bridge to LUTX | 12 (14.8%) |
|  | LAS | 35.5 [33.5 - 40.5] |
| Perioperative | Intraoperative ECMO | 39 (48.1%) |
|  | Postoperative ECMO | 18 (22.2%) |
|  | Blood components (L) | 1355 [570 - 2995] |
|  | Red Blood Cells (units) | 4 [2 - 7] |
| Donor | DBD donor (%) | 77 (95.1%) |
|  | Oto SCORE | 3 [1 - 4] |
|  | total warm-ischemia ime (min) | 152 [139 - 172] |
|  | total cold-ischemia time (min) | 871 [737 - 1095] |
|  | EVLP (%) | 15 (18.5%) |

*) Two patients with preoperative chronic kidney disease (i.e., eGFR < 60 mL/min/1.73 m^2^) were not included. BMI, body mass index; PAH, pulmonary arterial hypertension; FEdx, right ventricular ejection fraction; eGFR, estimated glomerular filtration rate; LUTX, lung transplant; LAS, lung allocation score; ECMO, extracorporeal membrane oxygenation; DBD, donation after brain death; EVLP, *ex-vivo* lung perfusion.

1. *Clinical Course of the two patients with pre-operative CKD*

Both patients were female and were aged 18 and 35 years old, respectively. Both were underweight (i.e., BMI 17.5 and 18.8 kg/m^2^, respectively), with a moderate-high LAS (i.e., 41.4 and 32.2, respectively). Baseline eGFR and serum creatinine for these patients were 35.7 and 6.2 mL/min/1.73 m^2^ and 1.6 and 10.2 mg/dL, respectively. Both needed ECMO during the LUTX procedure (the first as a bridge to LUTX). Neither of them underwent RRT prior to LUTX, but RRT was employed in both cases in the immediate postoperative period, resulting in serum creatinine concentrations of 0.99 and 2.78 mg/dL on postoperative day 1, peaking at 2.17 and 5.24 mg/dL during the ICU stay and ending up at 0.83 and 4.56 mg/dL at hospital discharge. At 1-year follow-up, both patients showed no improvement in renal function and they eventually died at 1752 days and 438 days after LUTX, prior to receiving a renal transplant.

1. **Figure S1. Changes in serum creatinine concentration at the different time points**. Box-plots (median and interquartile ranges) of creatinine levels. Black markers represent outliers. p < 0.05 vs. enrollment, pre-operative, postoperative day 1, and ICU discharge.
2.
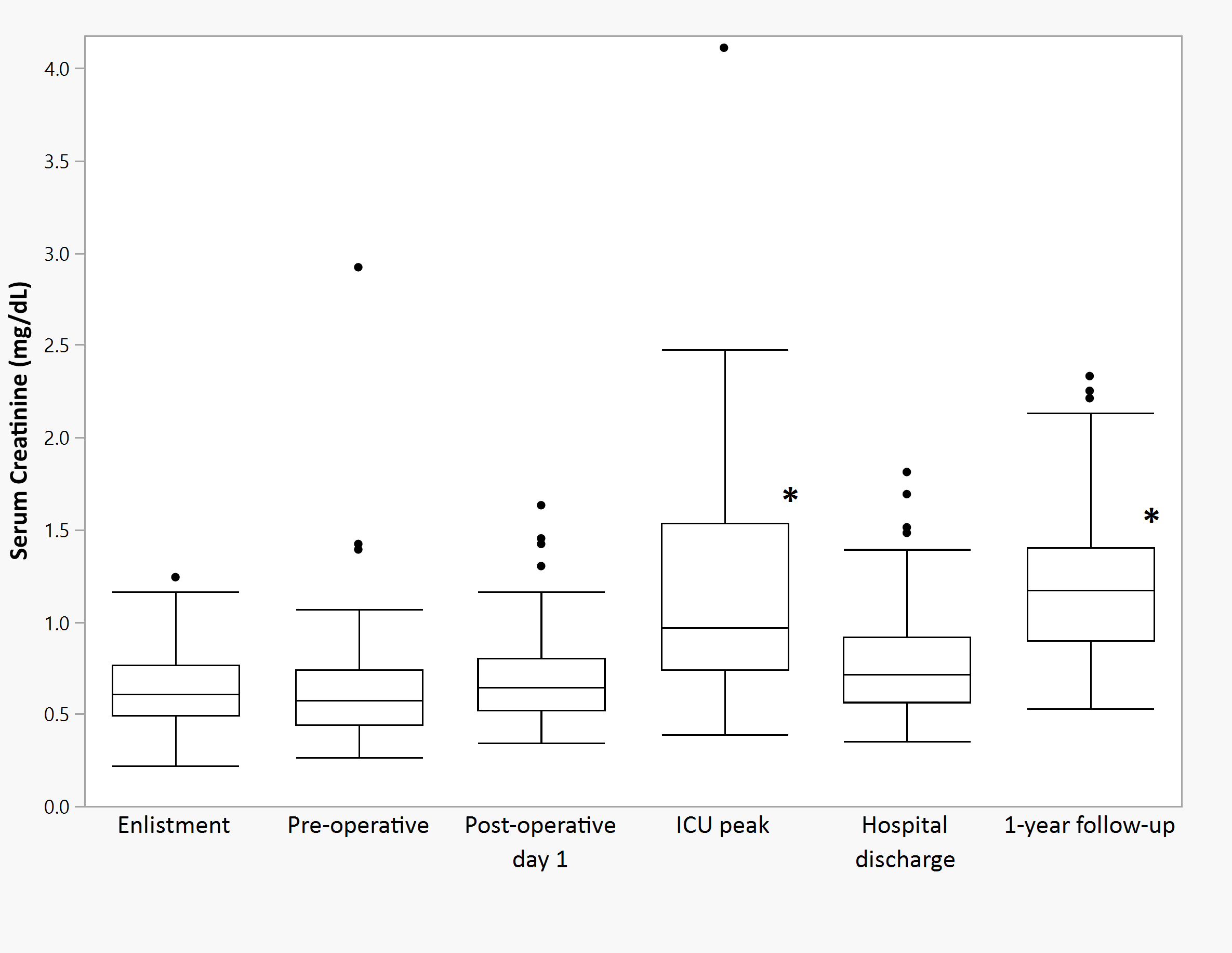
**Figure S2.. Impact of acute kidney injury stage during ICU stay upon short-term outcomes.**


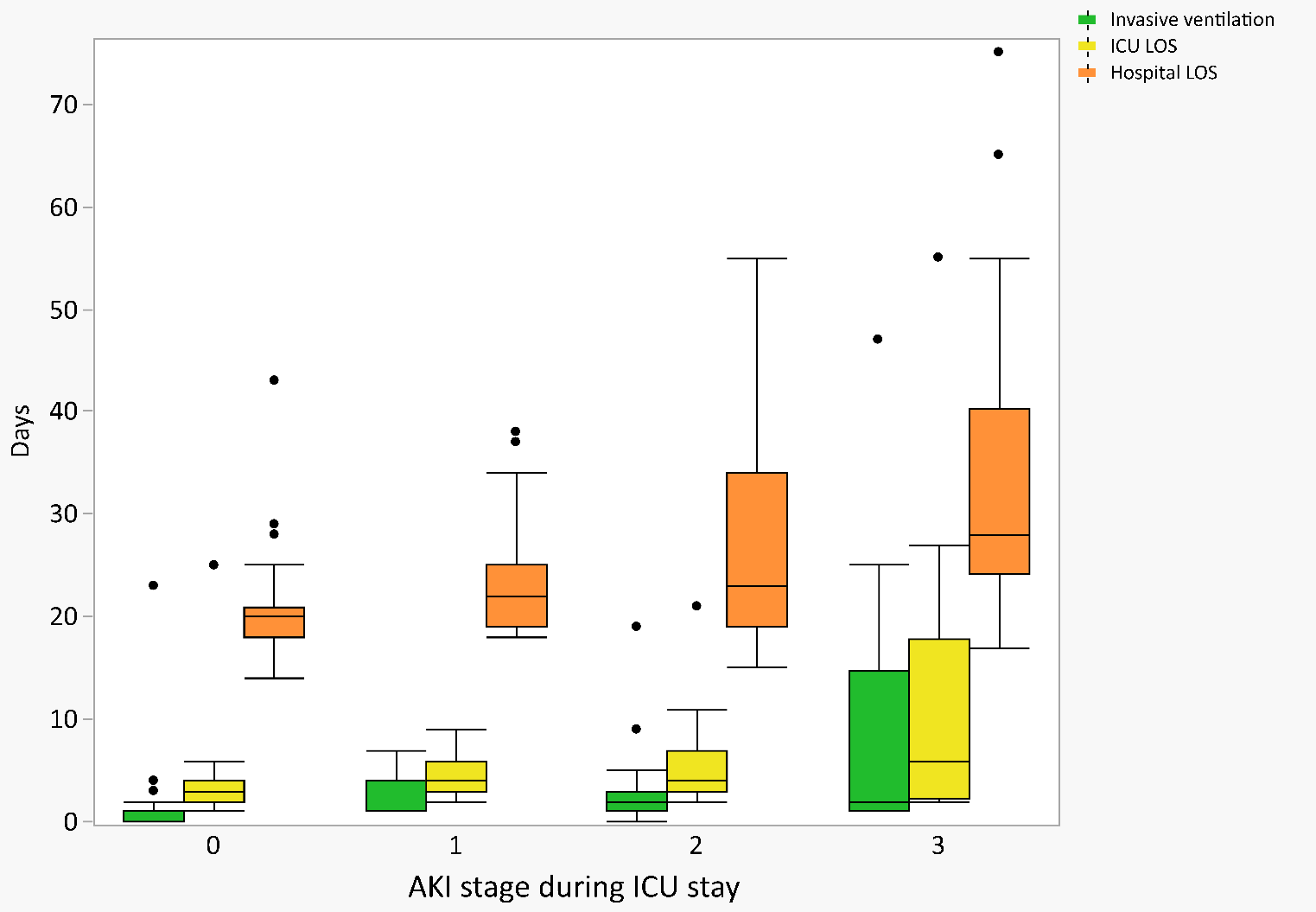
AKI, acute kidney injury; ICU, intensive care unit; LOS, length of stay.

**e) Figure 4.** **Mosaic plot of the incidence of 72-hour primary graft dysfunction vs. acute kidney injury during ICU stay.**
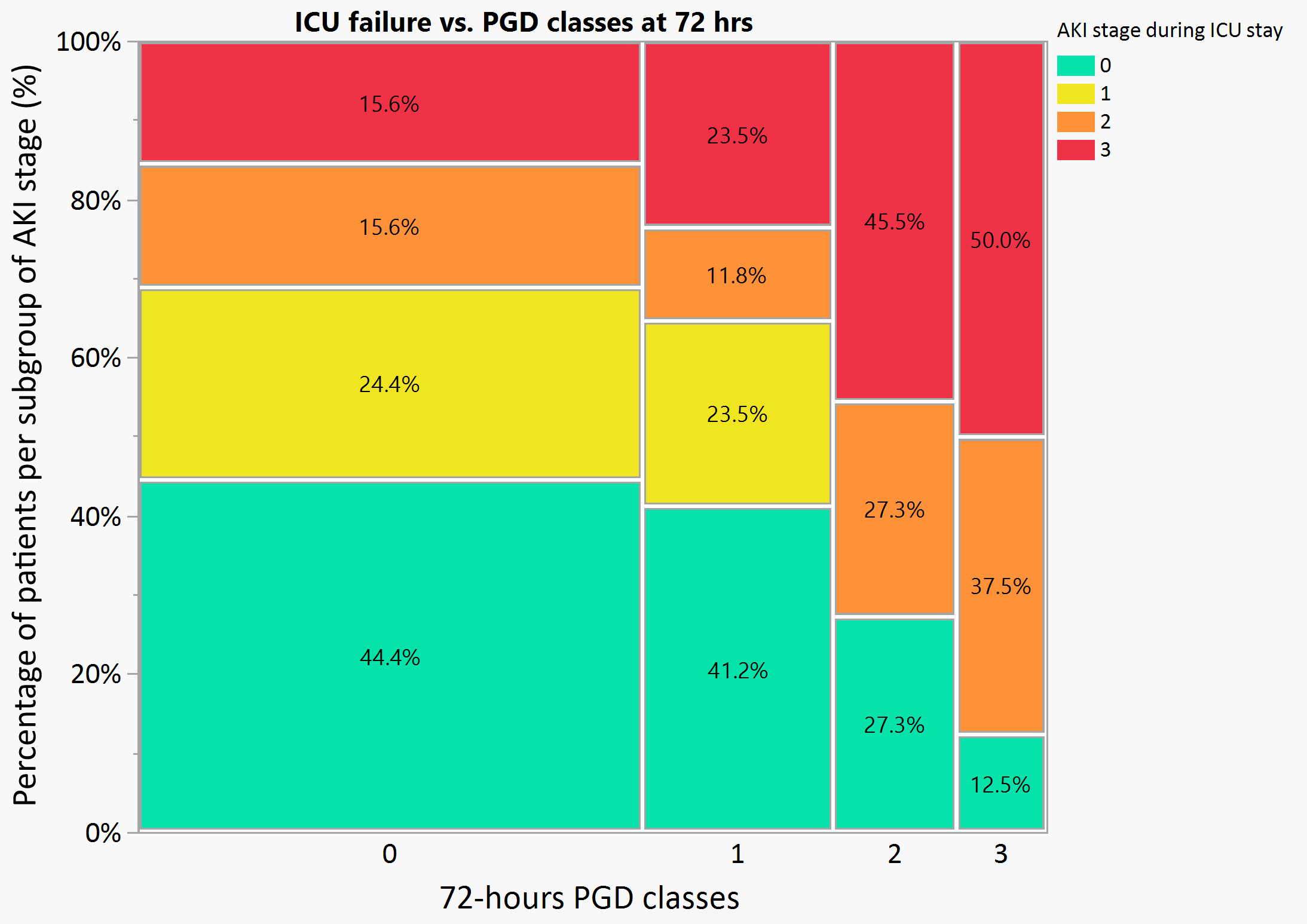


The width, height, and area of the rectangles are proportional to the number of patients per cohort, the frequency of acute kidney injury stage, and the cell frequencies of the contingency table. AKI, acute kidney injury; PGD, primary graft dysfunction; ICU, intensive care unit. Patients with pre-operative CKD (n=2) were excluded from the analysis.

1. **Table S2. Survival analysis.**

|  | p | HR | 95% CI |
| --- | --- | --- | --- |
| Age (years) | 0.059 | 0.931* | 0.854 – 1.001 |
| Sex (male) | 0.939 | 1.045 | 0.334 - 3.270 |
| BMI (kg/m2) | 0.968 | 1.053* | 0.069 - 12.614 |
| Diabetes (%) | 0.187 | 2.417 | 0.639 - 9.136 |
| LAS | 0.112 | 0.032* | 0.001 - 1.768 |
| PAH (%) | 0.751 | 0.791 | 0.182 – 3.438 |
| Intraoperative ECMO | 0.582 | 1.397 | 0.428 - 4.559 |
| AKI stage >= 1 | 0.015 | 5.392 | 1.106 - 26.273 |

HR, Hazard Ratio; CI, Confidence Interval; BMI, Body Mass Index; LAS, Lung Allocation Score; PAH, Pulmonary Arterial Hypertension; ECMO, Extracorporeal Membrane Oxygenation; AKI, Acute Kidney Injury. *) RR per unit change in regressor.

1. **Table S3.** **Risk factors for acute kidney injury (AKI stage ≥ 1 vs. AKI stage = 0).**

|  | **Clinical Characteristic** | **Acute kidney injury stage** | | | | **P*** | **OR (95% CI)**** |
| --- | --- | --- | --- | --- | --- | --- | --- |
|  |  | **0 (n=31, 38%)** | **1 (n=15, 18%)** | **2 (n=15, 18%)** | **3 (n=20, 27%)** |  |  |
| Enlistment | Age (years) | 28 [24 - 37] | 33 [22 - 42] | 26 [21 - 36] | 31 [23 - 37] | 0·884 | 0·99 (0·95-1·04) |
|  | Sex (male) | 20 (64·5%) | 8 (53·3%) | 4 (26·7%) | 8 (40·0%) | **0·040** | **0·36 (0·14-0·92)** |
|  | BMI (kg/m2) | 20·3 [18·4 - 21·8] | 20·5 [18·3 - 22·6] | 19·3 [17·9 - 21·1] | 19·1 [18·8 - 20·6] | 0·421 | 0·91 (0·74-1·13) |
|  | Diabetes (%) | 18 (58·1%) | 8 (53·3%) | 7 (46·7%) | 12 (60·0%) | 0·720 | 0·84 (0·34-2·09) |
|  | PAH (%) | 4 (12·9%) | 4 (28·6%) | 3 (20·0%) | 6 (31·6%) | 0·124 | 2·50 (0·73-8·56) |
|  | FEdx < 40% (%) | 5 (16·1%) | 2 (15·4%) | 2 (14·3%) | 5 (31·2%) | 0·600 | 1·37 (0·41-4·60) |
|  | eGFR | 125 [104 - 149] | 132 [110 - 178] | 121 [93 - 146] | 140 [101 - 171] | 0·211 | 1·00 (0·99-1·01) |
|  | Waiting list (days) | 113 [55-362] | 135 [49-244] | 182 [92-391] | 126 [39-402] | 0·809 | 0·99 (0·99-1·00) |
|  | Bridge to LUTX (%) | 1 (3·2%) | 2 (13·3%) | 1 (6·7%) | 8 (40·0%) | **0·011** | **8·46 (1·03-69·2)** |
|  | LAS | 35·3 [33·2 - 40·7] | 35·3 [33·6 - 43·4] | 34·2 [33·5 - 39·6] | 37·4 [34·3 - 49·7] | 0·077 | 1·04 (0·98-1·11) |
| Perioperative | Intraoperative ECMO | 13 (41·9%) | 6 (40·0%) | 6 (40·0%) | 14 (70·0%) | 0·949 | 0·97 (0·38-2·43) |
|  | Postoperative ECMO | 2 (6·4%) | 3 (20·0%) | 4 (26·7%) | 9 (45·0%) | **0·004** | **6·82 (1·44-32·1)** |
|  | Blood components (mL) | 1070 [570 - 1925] | 1140 [570 - 2995] | 1885 [285 - 4700] | 2370 [1400 - 4700] | **0·002** | **1·11 (1·01-1·21)** |
|  | Red Blood Cells (units) | 3 [1 - 5] | 4 [2 - 7] | 4 [1 - 10] | 6 [3 - 9·75] | **0·004** | **1·17 (1·01-1·35)** |
| Donor | DBD donor (%) | 30 (96·8%) | 14 (93·3%) | 14 (93·3%) | 19 (95·0%) | 0·564 | 0·52 (0·05-5·26) |
|  | oto SCORE | 2 [1 - 3·25] | 2·5 [1 - 5·25] | 3 [1 - 4·75] | 3 [1 - 4] | 0·110 | 1·17 (0·95-1·44) |
|  | total warm-ischemia time (min) | 152 [138 - 164] | 140 [120 - 177] | 158 [140 - 182] | 152 [141 - 177] | 0·365 | 1·00 (0·99-1·01) |
|  | total cold-ischemia time (min) | 827 [678 - 902] | 858 [762 - 1230] | 880 [620 - 1366] | 951 [836 - 1101] | **0·008** | **1·00 (1·00-1·01)** |
|  | EVLP (%) | 4 (12·9%) | 4 (26·7%) | 4 (26·7%) | 3 (15·0%) | 0·295 | 1·90 (0·54-5·25) |

BMI, body mass index; PAH, pulmonary arterial hypertension; FEdx, right ventricular ejection fraction; eGFR, estimated glomerular filtration rate; LUTX, lung transplant; LAS, lung allocation score; ECMO, extracorporeal membrane oxygenation; DBD, donation after brain death; EVLP, *ex-vivo* lung perfusion. *) AKI stage = 0 vs. AKI stage ≥ 1; **) OR, Odds Ratio estimated by the logistic regression model (ICU AKI stage = 0 vs. AKI stage ≥ 1). CI, confidence intervals. For continuous variables the OR per unit in change regressor is shown.

1. **Table S4. Risk factors for acute kidney disease.**

|  | **Clinical Characteristic** | **Acute kidney disease stages** | | | | **P*** | **OR (95% CI)**** |
| --- | --- | --- | --- | --- | --- | --- | --- |
|  |  | **0 (n=35, 38%)** | **1 (n=15, 18%)** | **2 (n=19, 18%)** | **3 (n=10, 27%)** |  |  |
| Enlistment | Age (years) | 28 [22 - 36] | 26 [22 - 36] | 35 [25 - 40] | 29 [21 - 37] | 0·307 | 1·02 (0·97-1·07) |
|  | Sex (male) | 24 (68·6%) | 6 (40·0%) | 8 (42·1%) | 2 (20·0%) | **0·014** | **0·31 (0·12-0·81)** |
|  | BMI (kg/m2) | 20·2 [18·4 - 22·1] | 18·7 [17·6 - 21·8] | 19·3 [18·8 - 21·5] | 19·4 [18·5 - 20·7] | 0·796 | 0·97 (0·78-1·20) |
|  | Diabetes (%) | 23 (65·7%) | 5 (33·3%) | 10 (52·6%) | 6 (60·0%) | 0·918 | 0·95 (0·38-2·34) |
|  | PAH (%) | 4 (11·7%) | 4 (26·7%) | 5 (26·3%) | 4 (44·4%) | 0·156 | 2·19 (0·73-6·51) |
|  | FEdx < 40% (%) | 8 (25·0%) | 0 (0·0%) | 5 (26·3%) | 1 (14·3%) | 0·669 | 1·29 (0·39-4·22) |
|  | eGFR | 131 [104 - 151] | 137 [98 - 154] | 121 [98 - 161] | 122 [97 - 147] | 0·405 | 0·99 (0·98-1·00) |
|  | Waiting list (days) | 135 [60-358] | 145 [57-223] | 180 [59-391] | 68 [17-317] | 0·690 | 0·99 (0·99-1·01) |
|  | Bridge to LUTX (%) | 1 (2·8%) | 3 (20·0%) | 2 (10·5%) | 5 (50·0%) | **0·030** | **4·00 (1·08-14·7)** |
|  | LAS | 34·5 [33·0 - 38·5] | 35·1 [34·0 - 41·2] | 37·1 [33·6 - 40·8] | 39·8 [35·2 - 65·3] | 0·073 | 1·03 (0·99-1·08) |
| Perioperative | Intraoperative ECMO | 19 (54·3%) | 12 (80·0%) | 13 (68·4%) | 5 (50·0%) | 0·949 | 1·03 (0·41-2·58) |
|  | Postoperative ECMO | 4 (11·4%) | 3 (20·0%) | 5 (26·3%) | 5 (50·0%) | **0·025** | **3·37 (1·14-10·0)** |
|  | Blood components (mL) | 1320 [570 - 2780] | 570 [285 - 2995] | 1925 [785 - 3890] | 2210 [1068 - 3041] | **0·014** | **1·05 (1·00-1·11)** |
|  | Red Blood Cells (units) | 4 [2 - 7] | 2 [1 - 3] | 4 [2 - 9] | 6 [3 - 8] | **0·011** | **1·10 (1·00-1·20)** |
| Donor | DBD donor (%) | 34 (97·1%) | 15 (100·0%) | 16 (84·2%) | 10 (100·0%) | 0·125 | 0·19 (0·01-1·91) |
|  | oto SCORE | 2·5 [1 - 5] | 2 [1 - 3] | 3 [1 - 4] | 4 [3 - 7] | 0·785 | 1·02 (0·85-1·24) |
|  | total warm-ischemia time (min) | 144 [131 - 166] | 152 [135 - 172] | 159 [140 - 176] | 155 [151 - 180] | 0·658 | 1·00 (0·99-1·01) |
|  | total cold-ischemia time (min) | 871 [757 - 1133] | 840 [678 - 1028] | 901 [615 - 1760] | 946 [633 - 1100] | **0·027** | **1·00 (1·00-1·01)** |
|  | EVLP (%) | 7 (20·0%) | 0 (0·0%) | 7 (36·8%) | 1 (10·0%) | 0·189 | 2·13 (0·68-6·63) |

BMI, body mass index; PAH, pulmonary arterial hypertension; FEdx, right ventricular ejection fraction; eGFR, estimated glomerular filtration rate; LUTX, lung transplant; LAS, lung allocation score; ECMO, extracorporeal membrane oxygenation; DBD, donation after brain death; EVLP, *ex-vivo* lung perfusion. The 2 patients who died at 90-day follow-up are not shown. *) AKD stage ≤ 1 vs. AKD stage > 1; **) OR, Odds Ratio estimated by the logistic regression model (AKD stage ≤ 1 vs. AKD stage > 1). CI, confidence intervals. For continuous variables the OR per unit in change regressor is shown.
